# Narrow-Linewidth GaN-on-Si Laser Diode with Slot Gratings

**DOI:** 10.3390/nano11113092

**Published:** 2021-11-16

**Authors:** Yongjun Tang, Meixin Feng, Jianxun Liu, Shizhao Fan, Xiujian Sun, Qian Sun, Shuming Zhang, Tong Liu, Yaping Kong, Zengli Huang, Masao Ikeda, Hui Yang

**Affiliations:** 1 School of Nano-Tech and Nano-Bionics, University of Science and Technology of China, Hefei 230026, China; yjtang2018@sinano.ac.cn (Y.T.); jxliu2018@sinano.ac.cn (J.L.); szfan2020@sinano.ac.cn (S.F.); smzhang2010@sinano.ac.cn (S.Z.); hyang2006@sinano.ac.cn (H.Y.); 2 Key Laboratory of Nanodevices and Applications, Suzhou Institute of Nano-Tech and Nano-Bionics (SINANO), Chinese Academy of Sciences (CAS), Suzhou 215123, China; xjsun2018@sinano.ac.cn (X.S.); mikeda2013@sinano.ac.cn (M.I.); 3 Guangdong (Foshan) Branch, SINANO, CAS, Foshan 528000, China; 4 Vacuum Interconnected Nanotech Workstation, SINANO, CAS, Suzhou 215123, China; tliu2015@sinano.ac.cn (T.L.); ypkong2020@sinano.ac.cn (Y.K.); zlhuang2008@sinano.ac.cn (Z.H.)

**Keywords:** narrow linewidth, GaN-on-Si, laser diode, III nitride photonics, etching damage

## Abstract

This letter reports room-temperature electrically pumped narrow-linewidth GaN-on-Si laser diodes. Unlike conventional distributed Bragg feedback laser diodes with hundreds of gratings, we employed only a few precisely defined slot gratings to narrow the linewidth and mitigate the negative effects of grating fabrication on the device performance. The slot gratings were incorporated into the ridge of conventional Fabry-Pérot cavity laser diodes. A subsequent wet etching in a tetramethyl ammonium hydroxide solution not only effectively removed the damages induced by the dry etching, but also converted the rough and tilted slot sidewalls into smooth and vertical ones. As a result, the threshold current was reduced by over 20%, and the reverse leakage current was decreased by over three orders of magnitude. Therefore, the room-temperature electrically pumped narrow-linewidth GaN-on-Si laser diode has been successfully demonstrated.

## 1. Introduction

Si photonics have been widely deemed as one of the leading technology paths for meeting the demand for a rapidly increasing data transfer rate, owing to the advantages of a large bandwidth, high speed and low power consumption [1,2,3,4,5]. However, the indirect bandgap nature of Si prohibits an efficient light emission [6,7]. Alternatively, direct-bandgap III-nitride semiconductors with a wide emission wavelength ranging from ultraviolet to infrared are the ideal materials for light-emitting diodes and laser diodes (LDs) [8,9,10]. Compared with conventional Fabry-Pérot (F-P) cavity LDs, narrow-linewidth LDs featured with precise wavelength tuning and high-speed modulation are more promising for optical interconnection [11]. Therefore, narrow-linewidth GaN-based LDs grown on Si could be utilized as a potential on-chip light source for Si photonics with III-nitride waveguides [12,13,14]. Moreover, narrow-linewidth GaN-based LDs also have emerging applications in atomic clocks, underwater communication, visible light communication, sensing, and master oscillator power amplifier systems [15,16,17,18,19,20,21].

The fabrication of narrow-linewidth GaN-based LDs faces multiple challenges. First of all, in order to narrow the lasing spectra, more than 100 pairs of Bragg gratings are often incorporated into conventional GaN-based F-P LDs [22,23,24]. Due to the chemical inertness of GaN, such Bragg gratings are usually fabricated by dry etching [25,26]. The sidewalls of the grating often suffer from roughness, poor steepness, and etching damage due to mask deformation, limitation of the aspect ratio, and high energy ion bombardment during the dry etching [27,28,29]. This would result in a large optical loss and low internal quantum efficiency owing to the light scattering and surface nonradiative recombination. Consequently, the threshold current of GaN-based narrow-linewidth LDs increased substantially, even by more than twice after the fabrication of Bragg gratings [30,31]. In addition, compared with that of GaAs or InP-based infrared lasers, the shorter emission wavelengths in GaN-based visible-light and ultraviolet LDs require smaller grating periods, generally in the sub-micrometer range. Therefore, the precise fabrication of such grating structures for GaN-based narrow-linewidth LDs is more challenging.

In this study, we report room-temperature electrically pumped narrow-linewidth GaN-on-Si LDs by mitigating the negative effects of slot etching. Only a few rationally designed slot gratings were introduced into the ridge of conventional F-P LDs to narrow the linewidth. A wet chemical treatment in a tetramethyl ammonium hydroxide (TMAH) solution effectively eliminated the damages caused by the dry etching, reducing the optical loss and electrical shunting. As a result, substantial reductions in the threshold current and leakage current were clearly observed.

## 2. Experimental Section

The slot and spacing are defined by *W_sl_* = (2*i* + 1)*λ*_0_/4*n_sl_* and *W_sp_ =* (2*j* + 1)*λ*_0_/4*n_sp_*, respectively, where *i* and *j* are integers, and *n_sl_* and *n_sp_* are the effective refractive indexes of the slot region and the spacing region, respectively. *λ*_0_ is the Bragg wavelength and set to be the lasing wavelength of GaN-on-Si F-P LD [32]. The Bragg equation can be expressed as 2(*n_sl_W_sl_* + *n_sp_W_sp_*) = *mλ*_0_, where *m* is the order of the Bragg grating. The *n_sl_* and *n_sp_* were 2.455 and 2.523, calculated by the finite difference beam propagation method. It is worth noting that while the slot width is much larger than the lasing wavelength, the light scattering loss would be largely increased due to optical diffraction [32,33]. As a compromise between the lasing wavelength (~410 nm) and the aspect ratio limitation of focused ion beam (FIB) technology, the slot width *W_sl_* was fixed as 380 nm to reduce light scattering loss, together with a spacing width (*W_sp_*) of 1680 nm.

**Figure 1:**
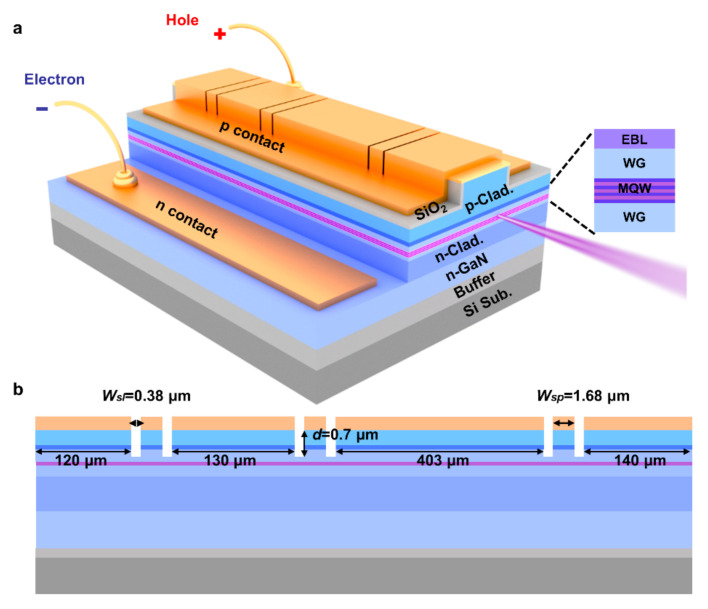
Schematic diagrams of the (**a**) side view and (**b**) cross-sectional view along the ridge of GaN-on-Si slotted F-P LD.

GaN-based laser material was epitaxially grown on an Si substrate by metalorganic chemical vapor deposition. Trimethylgallium, trimethylaluminum, trimethylindium and ammonia were used as precursors for gallium, aluminum, indium and nitrogen, respectively. The carrier gases are nitrogen and hydrogen. Monosilane and bisethylcyclopentadienylmagnesium were used as n- and p-type dopants, respectively. To eliminate the negative effect caused by the mismatch in both the lattice constant and coefficient of thermal expansion and condensed phases containing Si-basis [34,35], a carefully engineered AlN/AlGaN multilayer buffer was grown, and hence high-quality epitaxial GaN-on-Si templates could be obtained. On these templates, the laser structure consisted of an n-type GaN contact layer, an n-type Al_0.07_Ga_0.93_N cladding layer (CL), three pairs of In_0.12_Ga_0.88_N/In_0.02_Ga_0.98_N quantum wells (QWs) sandwiched by In_0.01_Ga_0.99_N waveguide layers (WG), an Al_0.2_Ga_0.8_N electron blocking layer (EBL), a p-type Al_0.075_Ga_0.925_N CL and a p-type GaN contact layer. More detailed laser structure information could be found in our previous work [36,37]. The as-grown laser epitaxial wafer was fabricated into edge-emitting F-P LDs with a ridge size of 4 × 800 μm^2^, and the cavity facets were formed by cleavage and left uncoated. Due to the highly resistive AlN/AlGaN buffer layer, a mesa was usually fabricated by dry etching to expose the n-GaN contact layer and form an n-type ohmic contact. Finally, a coplanar structure with both p- and n-contact pads on the same side was fabricated, as shown in Figure 1.

Subsequently, the slot gratings were formed in the ridge of the as-fabricated F-P LDs by FIB to narrow the linewidth, as shown in Figure 1a. Afterwards, the slotted F-P lasers were soaked in a 25% TMAH solution at 85 °C to remove the etching damages and steepen the sidewalls, and then an FIB etching together with scanning electron microscopy (SEM) measurement were used to track the slot morphology evolution. Before the FIB etching for the SEM observation, a layer of carbon was deposited on the slot surface to enhance the imaging contrast, and then a layer of platinum was also deposited to protect the surface from bombardment by a high energy ion beam.

Six slots divided the 800-μm long cavity into four sub-cavities with a length of 403, 120, 130 and 140 μm, respectively. The 403-μm long sub-cavity was mainly used to provide enough optical gain, and three shorter sub-cavities on both sides served as coupled cavities to select the optical mode based on the Vernier tuning mechanism [38,39,40]. From the viewpoint of optical coupling, it is preferable to fabricate slots through the QW to maximize the overlap between the optical field and slots. However, dry etching through the active region would significantly deteriorate the internal quantum efficiency. Therefore, the slots were etched with a depth of about 700 nm from the top p-GaN contact layer, and stopped at about 50 nm above the active region. The detailed information of the slotted F-P LD can be found in Figure 1b.

All the characterizations of GaN-on-Si LDs were performed at room temperature. The current-voltage (I-V) characteristic was measured by a continuous-wave power supply (Keithley 2400, Keithley, OH, USA), while the optoelectronic characteristics were tested under pulsed current injections with a 0.4% duty cycle and a 400 ns pulse width. The light output power as a function of the injection current (L-I) was measured by a calibrated optical power meter (PM121D, Thorlabs Inc., Newton, MA, USA). The electroluminescence (EL) spectra were collected by a high-resolution spectrometer (HORIBA 1250M, Horiba, Kyoto, Japan) with a resolution of approximately 6 pm. The morphologies of the slots and cavity facets were observed by SEM.

## 3. Results and Discussion

**Figure 2:**
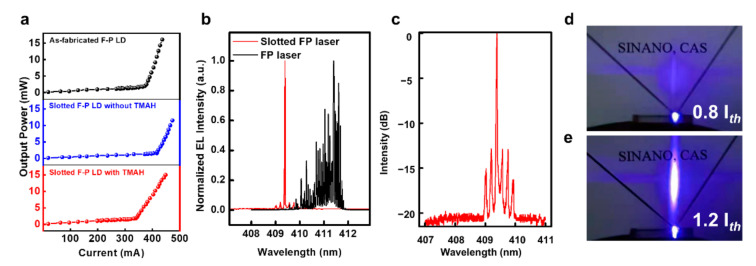
Characteristics of the GaN-on-Si LDs. (**a**) L-I curves of the as-fabricated F-P LD and slotted F-P LD with/without TMAH wet etching (Only one facet of the light was collected). (**b**) Lasing spectra of the slotted F-P LD and as-fabricated F-P LD. (**c**) Lasing spectrum of the slotted F-P LD and F-P LD in a semilog scale. (**d**,**e**) Far-field patterns of the slotted F-P LD (**d**) below the threshold current (0.8 *I_th_*) and (**e**) above the threshold current (1.2 *I_th_*).

Figure 2a shows the change in the L-I curve of the LD during the process flow. The threshold current was 375 mA for the as-fabricated F-P LD before etching slots and increased to 415 mA after the slot fabrication. The slightly increased (~11%) threshold current of the slotted lasers validated the efficacy of the rationally selected grating parameters, in stark contrast to the drastic increase of the threshold current reported in the literature [30,31]. In addition, the optical power of the slotted F-P LD (12.8 mW) decreased slightly when compared with the as-fabricated F-P LD (15.2 mW) at the injection current of 430 mA. This reduction in optical power after fabricating gratings is much less than that reported by other studies [30,31]. The superior device performance of the slotted F-P LDs was elucidated through two aspects. First, hundreds or thousands of gratings were usually adopted in the reported narrow-linewidth LDs to select the single longitudinal mode, and these gratings were often fabricated by dry etching, resulting in a large optical loss and low internal quantum efficiency [23,31]. However, for slotted F-P LDs, only several slot gratings were fabricated, which significantly reduced the etching damages. Second, by restricting the slots above the active region, we not only reduced the etching damage to the active region but also enabled a relatively uniform current injection across the slots, as the lateral diffusion length of holes is larger than half of the slot width [41,42]. Subsequently, we employed TMAH post-treatment to remove the dry-etching damages, reducing the threshold current of the slotted F-P LD from 415 to 340 mA, as shown in Figure 2a.

Figure 2b shows the EL spectra of the as-fabricated F-P LD and slotted F-P LD. The EL spectra of the slotted F-P laser under various injection currents can also be found in Supplementary Figure S1. Many longitudinal modes were clearly observed for the as-fabricated F-P LD with a side-mode suppression ratio (SMSR) of 0.16 dB, and the mode spacing was ~38 pm, corresponding to a cavity length of 800 μm. The full width at half maximum (FWHM) of the spectrum was more than 1 nm. In contrast, for the slotted F-P LD, only one dominant emission peak with an FWHM of 25 pm as well as several effectively suppressed longitudinal modes were observed. The FWHM is similar to that of distributed feedback lasers reported by other literature [25,30]. In Figure 2c, it is indicated that the SMSR was as high as 13 dB, which is comparable with the results reported in the literature [23,43]. The mode spacing was also increased to 180 pm, which was 4.7 times that of the conventional F-P LD. The peak wavelength of the slotted F-P LD was 409.4 nm and blue-shifted from that of the as-fabricated F-P LD (412.1 nm), which was mainly due to the mismatch between the Bragg wavelength and lasing wavelength. The far-field patterns of the slotted F-P LD at the injection current below and above the threshold current are presented in Figure 2d,e, respectively. Above the threshold current, a typical elliptical far-field pattern was clearly observed. The slotted structure only influences the longitudinal modes and has little effect on the transverse mode distribution. Therefore, the far-field pattern divergence angles should be similar to that of the as-fabricated GaN-on-Si F-P LD with the same epitaxial and ridge structure. The typical far-filed pattern divergence angles of GaN-on-Si F-P can be found in our previous work [36]. These results, together with the lasing spectrum and typical L-I curve, demonstrated the unambiguous electrically pumped lasing of the slotted F-P LD at room temperature.

**Figure 3:**
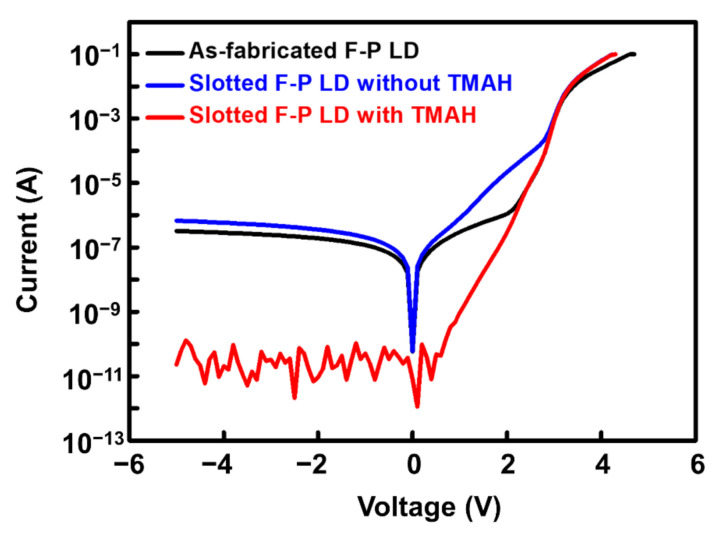
I-V curves of the as-fabricated F-P LD and slotted F-P LD with/without TMAH wet etching.

In order to investigate the effect of slot fabrication on the electrical properties of the LD, the I-V characteristics of as-fabricated F-P LD and slotted F-P with/without TMAH treatment were measured, as presented in Figure 3. It can be seen that after the slot fabrication in the ridge, the reverse leakage current was slightly increased from 3.2 × 10^−7^ to 6.7 × 10^−7^ A at a reverse bias of −5 V due to the etching damage, and the forward leakage current was also increased but was still very small when compared with the operation current. However, after the TMAH wet chemical etching, both the forward and reverse leakage currents of the slotted LDs were significantly decreased. In particular, the reverse leakage current was even reduced down to the measurement limitation of the source meter (~10^−11^ A). The reason will be discussed in the next section. All these observations clearly indicated that TMAH wet etching could significantly improve the laser performance.

**Figure 4:**
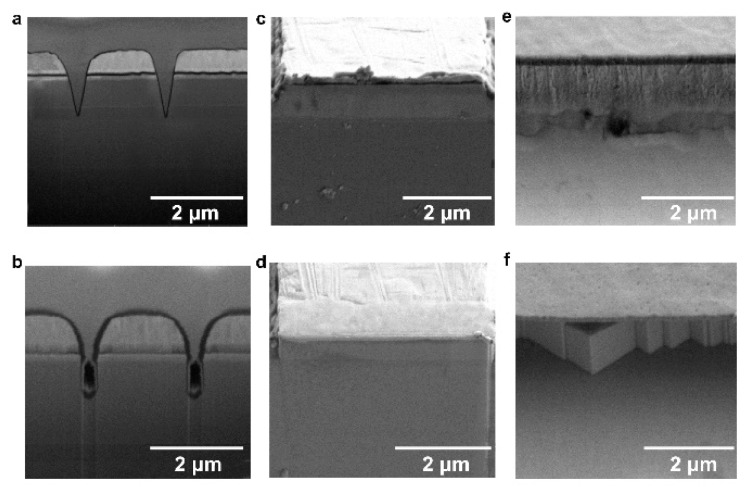
SEM images of the GaN-on-Si LDs, (**a**,**b**) cross-sectional images of the slots (**a**) before and (**b**) after the TMAH etching, (**c**,**d**) bird-eye view images of the uncoated cavity facet (**c**) before and (**d**) after the TMAH etching, (**e**,**f**) bird-eye view images of the mesa sidewalls formed by dry etching into n-GaN to fabricate a coplanar LD (**e**) before and (**f**) after the TMAH etching.

To shed light on the effects of the TMAH treatment, we conducted SEM observations on GaN-on-Si slotted F-P LDs. Figure 4a,b presents the cross-sectional images of the slots with/without TMAH polishing, respectively. Prior to TMAH wet etching, the slots exhibited V-shaped profiles and rough sidewalls, as shown in Figure 4a. This often induces optical loss and nonradiative recombination, causing an increase in the threshold current and a decrease in the slope efficiency, as shown in Figure 2a. Figure 4b shows that the TMAH wet etching transformed the slot profile from a V-shape to U-shape, and the sidewalls became steeper and smoother. Therefore, the slot sidewalls acted as better “optical mirrors” for the optical coupling and reflection. More importantly, the TMAH wet etching could also reduce the nonradiative recombination by removing the dry-etching damages.

The TMAH wet etching also made a distinct change in the cleaved cavity facets and the mesa sidewall. Figure 4c shows that the as-cleaved cavity facet was not smooth enough and was contaminated by particles during the cleavage, potentially due to the slight misorientation of the cleavage direction with the crystallographic m-plane. In Figure 4d, the uncoated cavity facet became smoother, and the absorbed particles were effectively removed by the TMAH wet etching, which increased the reflectivity and hence lowered the threshold current, while also reducing the slope efficiency, as shown in Figure 2a. Figure 4e,f shows the SEM images of the mesa sidewall before and after TMAH wet etching, respectively. Before wet etching, the mesa sidewall formed by dry etching suffered from etching damage and roughness, inducing a relatively high leakage current (~10^−7^ A) at −5 V. After TMAH polishing, the mesa sidewall was converted into the intersecting vertical and smooth m-planes, and the dry-etching damage was completely removed, which greatly reduced the leakage current by about three orders, down to ~10^−10^ A at −5 V. The decrease in the reverse bias current is common for all devices treated by TMAH. The statistical results are presented in Supplementary Figure S2.

The change in the morphology of the cavity facets and slot sidewalls caused by the TMAH etching originated from the anisotropic electrochemical properties of various crystallographic planes, which has been observed and explained in our previous work [44]. During the TMAH wet etching, Ga atoms in the m-plane surface with positively charged dangling bonds attract and react with OH^−^ in the TMAH solution, producing GaO_x_, which dissolves in the solution. The exposed N atoms with two negatively charged dangling bonds repel the OH^−^ anions and also prevent the underlying Ga atoms from being attacked by OH^−^. As a result, the wet etching ceased at the m-plane surface. Smooth and vertical m-plane sidewalls were obtained, and the dry etching-induced damages were also removed for the slots and cavity facets, which greatly improved the reflectivity and reduced the optical loss as well as the nonradiative recombination. On the other hand, in the case of the GaN a-plane mesa sidewalls, the TMAH wet chemical etching would also remove the dry-etching damage and then convert the sidewall surface into adjacent m-plane surfaces, intersecting at an angle of ~120°; eventually many tiny zig-zagged yet vertical m-plane mesa sidewalls were formed, as shown in Figure 4f, which eliminated the leakage path.

## 4. Conclusions

In summary, we have realized a room-temperature electrically pumped narrow-linewidth GaN-on-Si LD with an SMSR of ~13 dB. With several slots only, the rational design of the slot parameters enabled a narrow linewidth and minimal performance degradation, as compared to the conventional GaN-on-Si F-P LD. A substantial reduction in the threshold current and leakage current of the slotted F-P LD was observed after the TMAH etching, due to the removal of the dry etching-induced damages and the improvement in the sidewall. With a further improvement in the material quality and further optimization of the slot parameters to boost the optical feedback, the realization of low-threshold-current and narrow-linewidth GaN-on-Si LDs with a higher SMSR is very promising, which could be a potential on-chip light source for monolithically integrated Si photonics.

## Data Availability

The data presented in this study are available on request from the corresponding author.

## Supplementary Materials

The following are available online at https://www.mdpi.com/article/10.3390/nano11113092/s1, Figure S1 shows the electroluminescence spectra of the slotted F-P laser measured under various pulsed injection currents; Figure S2 shows statistical results in leakage current evolution.
