# Supplementary material for: Narrow-Linewidth GaN-on-Si Laser Diode with Slot Gratings
Source: Nanomaterials (Basel). 2021 Nov 16;11(11):3092. doi: 10.3390/nano11113092 (PMC8619470; doi:10.3390/nano11113092)
Supplement: Supplementary file 1 [file nanomaterials-11-03092-s001.zip › nanomaterials-1426123-supplementary.pdf]

nanomaterials

Supplementary Materials

Narrow-linewidth GaN-on-Si laser diode with slot gratings

Yongjun Tang[1,2], Meixin Feng[1,2,3*], Jianxun Liu[1,2,3], Shizhao Fan[1,2], Xiujian Sun[2,3], Qian Sun[1,2,3*], Shuming Zhang[1,2,3], Tong Liu[4], Yaping Kong[4], Zengli Huang[4], Masao Ikeda[2] and Hui Yang1[,2,3,4]

1 School of Nano-Tech and Nano-Bionics, University of Science and Technology of China, Hefei 230026, China
2 Key Laboratory of Nanodevices and Applications, Suzhou Institute of Nano-Tech and Nano-Bionics (SINANO), Chinese Academy of Sciences (CAS), Suzhou 215123, China
3 Guangdong (Foshan) Branch, SINANO, CAS, Foshan 528000, China
4 Vacuum Interconnected Nanotech Workstation, SINANO, CAS, Suzhou 215123, China
* Correspondence: Correspondence: mxfeng2011@sinano.ac.cn (M.F), qsun2011@sinano.ac.cn (Q.S)

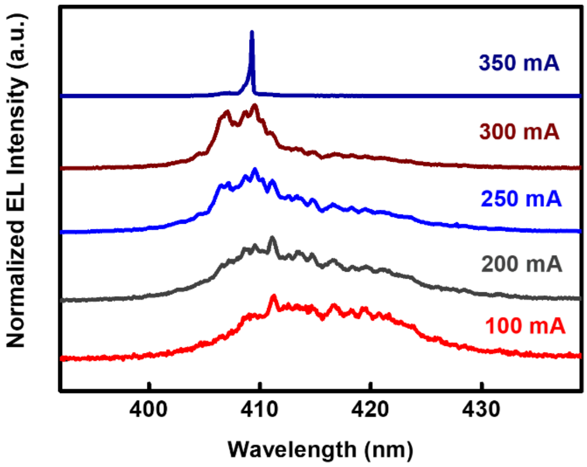

Figure S1. Electroluminescence spectra of the slotted F-P laser measured under various pulsed injection currents (collected by a fiber optic spectrometer (IdeaOptics FX4000)).

Figure S2 presents the electroluminescence spectra of the slotted LD under various pulsed injection currents at room temperature. At a low operation current, the spontaneous emission was dominant, hence several broad electroluminescence spectra were collected. As the injection current was incerased to 350 mA, the electroluminescence spectrum was quickly narrowed down, and the stimulated radiation was dominant.

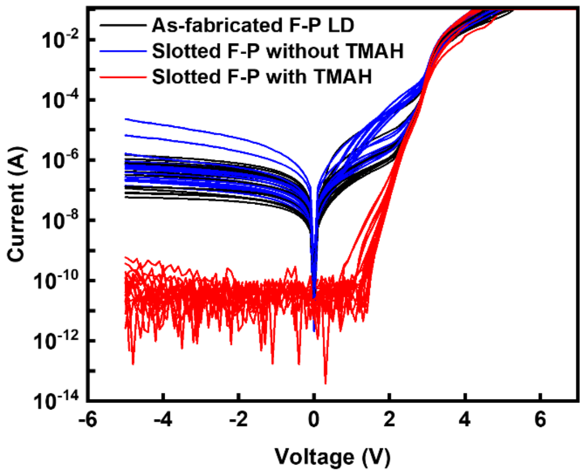

Figure S2. I-V curves of GaN-on-Si as-fabricated F-P LD and slotted F-P LD with/without TMAH wet etching.

Due to the highly resistive AlN/AlGaN buffer layer, a mesa was usually fabricated by dry etching to expose n-GaN contact layer and form n-type ohmic contact. Finally, a coplanar structure with both p- and n-contact pads at the same side was fabricated. Before TMAH wet etching, the mesa sidewall was suffered from dry-etching damage and roughness, inducing a relatively high leakage current. After TMAH polishing, the dry-etching damage was completely removed, and the mesa sidewall was converted into the intersecting vertical and smooth m-planes, which greatly reduced the leakage current, as shown in Figure 4.
